# Effectiveness of Acute Geriatric Unit Care Using Acute Care for Elders Components: A Systematic Review and Meta-Analysis

**DOI:** 10.1111/jgs.12028

**Published:** 2012-11-23

**Authors:** Mary T Fox, Malini Persaud, Ilo Maimets, Kelly O'Brien, Dina Brooks, Deborah Tregunno, Ellen Schraa

**Affiliations:** * School of Nursing, York UniversityToronto, Ontario, Canada; † School of Steacie Science and Engineering Library, York UniversityToronto, Ontario, Canada; ‡ Department of Physical Therapy, Graduate Department of Rehabilitation Sciences, University of TorontoToronto, Ontario, Canada; § School of Health Policy and Management, Faculty of Health, York UniversityToronto, Ontario, Canada

**Keywords:** ACE model, elderly, meta-analysis, function-focused interventions, acute geriatric unit

## Abstract

**Objectives:**

To compare the effectiveness of acute geriatric unit care, based on all or part of the Acute Care for Elders (ACE) model and introduced in the acute phase of illness or injury, with that of usual care.

**Design:**

Systematic review and meta-analysis of 13 randomized controlled and quasi-experimental trials with parallel comparison groups retrieved from multiple sources.

**Setting:**

Acute care geriatric and nongeriatric hospital units.

**Participants:**

Acutely ill or injured adults (N = 6,839) with an average age of 81.

**Interventions:**

Acute geriatric unit care characterized by one or more ACE components: patient-centered care, frequent medical review, early rehabilitation, early discharge planning, prepared environment.

**Measurements:**

Falls, pressure ulcers, delirium, functional decline at discharge from baseline 2-week prehospital and hospital admission statuses, length of hospital stay, discharge destination (home or nursing home), mortality, costs, and hospital readmissions.

**Results:**

Acute geriatric unit care was associated with fewer falls (risk ratio (RR) = 0.51, 95% confidence interval (CI) = 0.29–0.88), less delirium (RR = 0.73, 95% CI = 0.61–0.88), less functional decline at discharge from baseline 2-week prehospital admission status (RR = 0.87, 95% CI = 0.78–0.97), shorter length of hospital stay (weighted mean difference (WMD) = −0.61, 95% CI = −1.16 to −0.05), fewer discharges to a nursing home (RR = 0.82, 95% CI = 0.68–0.99), lower costs (WMD = −$245.80, 95% CI = −$446.23 to −$45.38), and more discharges to home (RR = 1.05, 95% CI = 1.01–1.10). A nonsignificant trend toward fewer pressure ulcers was observed. No differences were found in functional decline between baseline hospital admission status and discharge, mortality, or hospital readmissions.

**Conclusion:**

Acute geriatric unit care, based on all or part of the ACE model and introduced during the acute phase of older adults' illness or injury, improves patient- and system-level outcomes.

Adults aged 65 and older constitute the “core business” of hospitals.^1^ Although they represent 13% of the population in the United States^2^ and 14% of the population in Canada,^3^ older adults account for 43% of inpatient hospital days in the United States^4^ and 40% in Canada.^5^ This trend is likely to continue given population aging.^3^

During hospitalization for an acute event such as illness or injury, older adults are at risk of experiencing functional decline and iatrogenic complications, including falls, pressure ulcers, and delirium, which further contribute to functional decline.^6^ Hospital-acquired functional decline is associated with greater hospital expenditures, institutionalization, and mortality in older adults^7^ even after controlling for comorbidity and illness severity.^8^ Therefore, early intervention (before an acute episode is resolved) is critical because of the short length of time during which older persons can recover functional losses, resume their former lives, and avoid institutionalization.^9^

Dedicated geriatric units, based on a prehabilitation^10^ and function-focused^11^ model of care called Acute Care for Elders (ACE), have been designed specifically to prevent functional decline and related complications in older adults admitted to the hospital for an acute event.^12^,^13^ In response to an increasingly older and complex hospital population, some service providers have adopted the ACE model on hospital units where older adults are admitted.^13^ However, the overall effect of acute geriatric unit care, based on all or part of the ACE model and introduced during the acute phase of illness or injury, is unclear and unquantified.

Two systematic reviews of acute geriatric unit care based on the ACE model have been conducted,^14^,^15^ but the authors did not present results of meta-analyses, supporting the need for this current review. Three prior reviews combined data from studies conducted with individuals in the acute and subacute illness phases;^14^,^16^,^17^ the results have limited validity for individuals in the acute phase of an illness or injury. One meta-analysis^18^ imputed means for missing standard deviations for cost and length-of-stay outcomes in almost 30% of included studies,^18^ which may have resulted in an underestimation of the overall effect. Last, no meta-analysis of acute geriatric unit care included iatrogenic complications, which are critical indicators of quality hospital care.^19^

The purpose of this study was to determine the effectiveness of acute geriatric unit care, based on all or part of the ACE model components and introduced in the acute phase of illness or injury, in reducing iatrogenic complications, functional decline, length of hospital stay, poor discharge destination outcomes, mortality, costs, and hospital readmissions in older adults.

## Methods

A systematic review was performed that compared acute geriatric unit care, in which all or part of the ACE model components were introduced in the acute phase of illness or injury, with usual care using the Cochrane Collaboration Protocol.^20^

### Eligibility Criteria

Eligible studies included published and unpublished randomized controlled and quasi-experimental trials with parallel controls that compared acute geriatric unit care with usual care for adults aged 65 and older in the acute illness or injury phase.^17^ Acute geriatric unit care included at least one of the five ACE model components or principles:^13^,^21^,^22^ patient-centered care, defined as care activities (assessments and protocols) to prevent declines in activities of daily living (ADLs), mobility, continence, nutrition, skin integrity, mood, sleep, and cognition; frequent medical review, defined as activities to minimize the adverse effects of treatments on older adults' functioning; early rehabilitation, defined as the participation of physical or occupational therapists in daily team meetings for the purposes of initiating rehabilitation or standard provision of physical or occupational therapy; early discharge planning, defined as activities to facilitate return to the community; and prepared environment, defined as environmental modifications to facilitate physical and cognitive functioning. Usual care was defined as any care not provided on an acute geriatric unit.

Eligible studies included at least one primary (iatrogenic complications or functional decline) or secondary (length of hospital stay, discharge destination, mortality, costs, or hospital readmissions) outcome. Iatrogenic complications included falls (defined as the number of individuals who experienced ≥1 falls), pressure ulcers (defined as the number of individuals who experienced skin breakdown), or delirium (defined as the number of individuals diagnosed with ≥1 delirium episodes) during hospitalization. Functional decline was defined as loss of independence at discharge in at least one of five basic ADLs: transfers, toileting, dressing, eating, or bathing,^10^ as measured as the Barthel Index or the Katz ADL scale, 2 weeks before hospital admission or upon hospital admission. Length of hospital stay was defined as the total number of days in the hospital or as the time between study admission and discharge if total number of days in the hospital was not provided. Discharge destination included discharge to home (defined as own home or with family) or nursing home (defined as nursing home, sheltered living, or hostel). Mortality refers to number of deaths during hospitalization. Costs were defined as total hospital costs associated with care for the duration of hospital stay. Cost data were standardized to U.S. dollars for a common price year of 2000, the last study year with published cost data. Hospital readmissions refer to the number of individuals readmitted one or more times to an acute care hospital within 1 or 3 months after discharge from the study hospital.

Studies unavailable in English or French, involving individuals undergoing elective surgical procedures or receiving palliative care, including social admissions, or with historical control groups were ineligible.

### Search Strategy and Study Selection

An information specialist conducted the literature search with input from team members with expertise in the clinical area to identify keywords reflective of the ACE model (Appendix S1 of the electronic supplementary material). Electronic databases searched were as follows: Evidence-Based Medicine Reviews consisting of the Cochrane Library, DARE, HTA, NHSEED and ACP; MEDLINE; EMBASE; CINAHL; Proquest Dissertations and Theses; PubMed; Web of Science; SciSearch; PEDro; Sigma Theta Tau International's registry of nursing research; Joanna Briggs Institute; CRISP; and OT Seeker. Internet search engines included Google, Yahoo, Scirus, Healia, and HON. Hand-searching was conducted in the *Gerontologist*, *Age and Ageing*, *JAMA*, and bibliographies of all included articles and previous systematic reviews.

Two reviewers independently screened abstracts of the retrieved citations for potential inclusion. Disagreements about eligibility were resolved by consensus between two reviewers. Where consensus could not be reached, a third team member independently reviewed the abstract and determined final inclusion. When necessary, the complete article was reviewed to determine eligibility.

### Data Extraction and Risk of Bias Assessment

Two reviewers independently extracted relevant data from each included article and entered the data into a standardized data extraction form. Information categories included study design, participants, ACE components, healthcare providers, occasions of measurement, and outcomes. Two reviewers independently assessed each study's risk of bias using six defined domains: sequence generation; allocation concealment; blinding of participants, personnel, and outcome assessors; completeness of outcome data; selective reporting; and other sources of bias.^20^

Study authors were contacted if additional data were required. Disagreements on data extraction and risk of bias assessments were resolved by consensus with assistance of a third team member.

### Data Analysis

When sufficient data were available and studies were comparable in terms of outcomes, meta-analyses were performed using review manager software.^20^ When data were neither retrievable from study authors nor derivable from available data, values were not assumed for the purposes of meta-analyses. In studies in which per-protocol and intention-to-treat data were reported, the latter were analyzed. Continuous and dichotomous outcomes were analyzed using a random-effects model to calculate weighted mean differences (WMDs) and risk ratios (RRs), respectively, with 95% confidence intervals (CIs). *P* < .05 was considered statistically significant for an overall effect. *P* < .10 was considered statistically significant for heterogeneity.^23^ Degree of heterogeneity is reported according to the I^2^ statistic, which refers to the degree of variation between studies.^20^ Because of the potential for clinical heterogeneity of study populations and ACE components and the associated risk of a false-negative I^2^ statistic,^24^ the CIs of individual studies contained in the forest plots were also examined.^20^ In situations in which heterogeneity was statistically significant or was not statistically significant but there was minimal overlap of the CIs, sensitivity analyses were performed whereby studies were systematically removed from meta-analyses to determine robustness of findings. Decisions for removing studies were based on their potential sources of variability; studies conducted on surgical units were removed first, followed by studies conducted on medical–surgical units, and then studies that did not implement all five ACE components, beginning with studies that implemented the fewest components.

## Results

### Description of Studies

**Figure 1:**
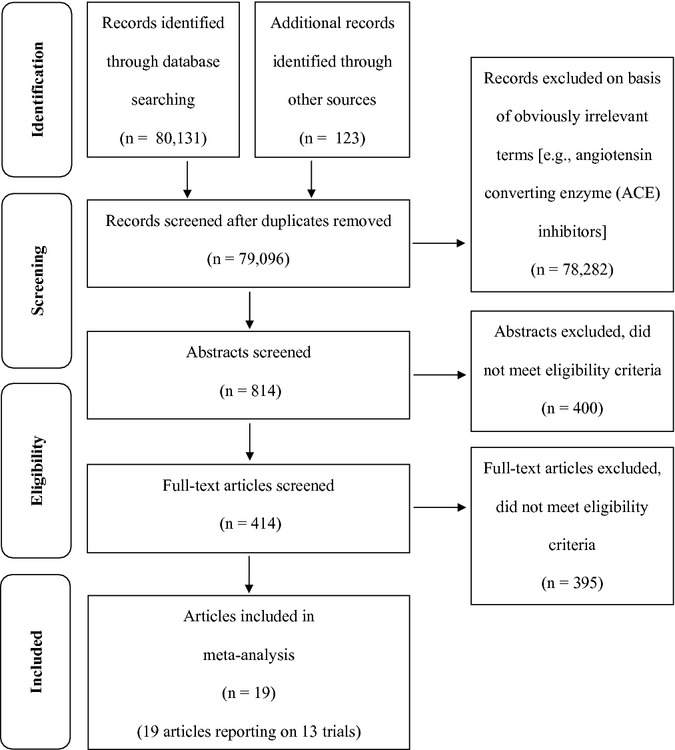
PRISMA flow diagram.^49^

Searches of all sources yielded 79,096 citations, of which 19 studies^21^,^22^,^25^–^41^ reporting on 13 trials met the inclusion criteria (Figure 1). Characteristics of the 13 trials^21^,^22^,^25^,^26^,^28^–^31^,^35^,^38^–^41^ are provided in Appendix S2 Table S1 of the electronic supplementary material.

Six thousand eight hundred thirty-nine participants were included in this review. The average study participant was aged 81, female (61.8%), and admitted with an acute medical illness (81.3%),^21^,^22^,^25^,^26^,^30^,^35^,^38^–^41^ fracture (6.9%),^26^,^29^,^31^ unspecified acute or critical illness (11.8%),^26^,^28^,^38^ or other morbidity.^21^,^22^,^25^,^29^–^31^,^35^,^38^–^40^ Sixty-two percent of the studies restricted eligibility to individuals admitted through hospital emergency departments.^21^,^22^,^25^,^29^–^31^,^40^,^41^

Acute geriatric unit care most often included patient-centered care,^21^,^22^,^25^,^26^,^28^–^31^,^35^,^38^–^41^ followed by frequent medical review,^21^,^22^,^25^,^28^,^31^,^35^,^39^–^41^ early rehabilitation,^21^,^22^,^25^,^28^,^31^,^40^,^41^ early discharge planning,^21^,^22^,^25^,^26^,^35^,^40^,^41^ and prepared environment.^21^,^22^,^35^,^39^,^41^ Acute geriatric unit teams comprised predominantly physicians and nurses,^21^,^22^,^25^,^26^,^28^–^31^,^35^,^38^–^41^ followed by physical therapists,^21^,^22^,^25^,^26^,^28^–^31^,^35^,^40^,^41^ social workers,^21^,^22^,^26^,^28^,^30^,^31^,^35^,^38^,^40^,^41^ geriatricians,^21^,^25^,^29^–^31^,^38^,^39^,^41^ and occupational therapists.^21^,^22^,^25^,^26^,^30^,^31^,^40^ Interdisciplinary teams met regularly to plan patient care.^21^,^22^,^26^,^28^,^29^,^31^,^35^,^39^–^41^

Usual care consisted of standard nursing and medical care that was neither functionally focused^21^,^22^ nor interdisciplinary team directed.^21^,^22^,^25^,^31^,^41^ Usual care was provided on medical,^21^,^22^,^25^,^30^,^35^,^39^–^41^ medical–surgical,^26^,^28^,^30^ or surgical orthopedic^29^,^31^ units.

#### Risk of Bias

Selection bias resulting from inadequate sequence generation was low in seven of the 13 studies.^21^,^22^,^25^,^26^,^30^,^31^,^41^ Three studies that used randomization provided insufficient information to draw conclusions in this domain^28^,^35^ or were considered not to have been properly randomized.^29^ Three studies were determined to have high risk of selection bias because randomization was not performed.^38^–^40^

Risk of selection bias resulting from inadequate allocation concealment was low in six of the 13 studies.^21^,^22^,^25^,^26^,^31^,^41^ Allocation was not concealed in one study, resulting in a high risk assessment.^30^ In all other studies, risk of bias was unclear because allocation information was not provided.^28^,^29^,^35^,^38^–^40^

Risk of performance bias related to double blinding (participants and personnel) was unclear because seven studies did not provide this information.^22^,^28^,^29^,^35^,^38^–^40^ Three studies were double blinded and considered to have low risk of performance bias,^21^,^26^,^31^ whereas three studies were not double blinded and were considered to have high risk of performance bias.^25^,^30^,^41^

Risk of detection bias related to blinding of outcome assessors was unclear because 11 studies did not provide this information.^21^,^22^,^25^,^26^,^28^–^30^,^35^,^38^–^40^ One study was considered high risk because outcomes assessors were blinded to only one of several outcomes.^41^ Only one study reported that outcome assessors were blinded and was considered to have low risk of bias.^31^

Risk of attrition bias related to completeness of outcome data was low in six studies^22^,^28^–^31^,^41^ and unclear in one.^38^ Six studies were considered to have high risk of bias because of postrandomization exclusions^25^,^39^ or attrition.^21^,^26^,^35^,^40^

Risk of reporting bias due to selective reporting was low^22^,^28^,^31^,^35^,^41^ or unclear.^25^,^29^,^30^,^38^–^40^ One study^26^ was considered to have high risk of reporting bias because its length-of-stay and cost data were missing. None of the 13 studies appeared to be at risk of other sources of bias that were not addressed in prior domains.

#### Effectiveness of Acute Geriatric Unit Care

Eleven meta-analyses were performed. Unpublished data were obtained from study authors to perform meta-analyses on functional decline between baseline 2-week prehospital admission status and discharge,^39^ length of hospital stay,^27^,^29^,^39^,^41^ mortality,^35^ and costs.^21^,^41^ Four sensitivity analyses were conducted for functional decline between baseline hospital admission status and discharge, length of hospital stay, discharge to nursing home, and costs.

#### Iatrogenic Complications

Falls and pressure ulcers were reported in the same two studies^26^,^31^ resulting in two meta-analyses. Acute geriatric unit care was associated with significantly fewer falls (RR = 0.51, 95% CI = 0.29–0.88; *P* = .02) and nonsignificantly fewer pressure ulcers (RR = 0.49, 95% CI = 0.23–1.04; *P* = .06) in acutely ill or injured older adults than usual care.

Delirium was reported in three studies.^25^,^31^,^39^ Meta-analysis of these three studies showed that acute geriatric unit care was associated with significantly less occurrence of delirium than usual care in acutely ill or injured older adults (RR = 0.73, 95% CI = 0.61–0.88; *P* = .001).

#### Functional Decline

Functional decline between baseline 2-week prehospital admission status and discharge was reported in six studies.^21^,^22^,^31^,^39^–^41^ Meta-analysis of these six studies indicated that individuals receiving acute geriatric unit care were 13% significantly less likely to experience functional decline between their baseline 2-week prehospital admission status and discharge than those receiving usual care (RR = 0.87, 95% CI = 0.78–0.97; *P* = .01).

Functional decline between baseline hospital admission status and discharge was reported in four studies.^21^,^22^,^40^,^41^ Meta-analysis of these four studies showed that, compared to usual care, individuals receiving acute geriatric unit care experienced no significant difference in risk of functional decline between baseline hospital admission status and discharge (RR = 0.83, 95% CI = 0.64–1.08; *P* = .16). Significant statistical heterogeneity was observed for this comparison. With removal of one outlier study^40^ during sensitivity analysis, statistical heterogeneity was resolved, although the effect remained nonsignificant (Table 1).

**Table 1:** Results of Meta-Analyses

Outcome	Individual Studies Included in Meta-Analysis	N	WMD (95% CI) or RR (95% CI)^a^	Test for Overall Effect, Z (*P-*Value)	I^2^ Statistic (*P-*Value) for Heterogeneity
Iatrogenic complications					
Falls	Collard et al.^26^ Olofsson^31^	749	0.51 (0.29–0.88)	2.41 (.02)	0% (.55)
Pressure ulcers	Collard et al.^26^ Olofsson^31^	749	0.49 (0.23–1.04)	1.87 (.06)	25% (.26)
Delirium	Asplund et al.^25^ Olofsson^31^ Vidan et al.^39^	1,154	0.73 (0.61–0.88)	3.29 (<.001)	0% (.44)
Functional decline at discharge from baseline					
2-week prehospital admission status	Barnes et al.^41^ Counsell et al.^21^ Landefeld et al.^22^ Olofsson^31^ Vidan et al.^39^ Zelada et al.^40^	4,485	0.87 (0.78–0.97)	2.55 (.01)	37% (.16)
Hospital admission status	Barnes et al.^41^ Counsell et al.^21^ Landefeld et al.^22^ Zelada et al.^40^	3,860	0.83 (0.64–1.08)	1.41 (.16)	68% (.03)
Outlier removed	Barnes et al.^41^ Counsell et al.^21^ Landefeld et al.^22^	3,717	0.92 (0.75–1.13)	0.83 (.41)	52% (.12)
Length of hospital stay, days^c^	Asplund et al.^25^ Barnes et al.^41^ Counsell et al.^21^ Covinsky et al.^27^, ^b^ Fretwell et al.^28^ González-Montalvo et al.^29^ Harris et al.^30^ Olofsson^31^ Stewart et al.^38^ Vidan et al.^39^ Zelada et al.^40^	6,098	–1.28 (–2.33 to –0.22)	2.37 (.02)	87% (<.001)
Outliers removed	Barnes et al.^41^ Counsell et al.^21^ Covinsky et al.^27^, ^b^ Zelada et al.^40^	3,956	–0.61 (–1.16 to –0.05)	2.12 (.03)	45% (.14)
Discharge destination					
Home	Asplund et al.^25^ Barnes et al.^41^ Collard et al.^26^ Fretwell et al.^28^ González-Montalvo et al.^29^ Harris et al.^30^ Landefeld et al.^22^ Olofsson^31^ Somme et al.^35^	4,315	1.05 (1.01–1.10)	2.69 (.01)	0% (.54)
Nursing home	Asplund et al.^25^ Collard et al.^26^ Counsell et al.^21^ Fretwell et al.^28^ González-Montalvo et al.^29^ Harris et al.^30^	3,378	0.96 (0.80–1.15)	0.48 (.63)	50% (.06)
Outliers removed	Asplund et al.^25^ Counsell et al.^21^ Harris et al.^30^	2,040	0.82 (0.68–0.99)	2.10 (.04)	0% (.57)
Mortality	Asplund et al.^25^ Barnes et al.^41^ Collard et al.^26^ Counsell et al.^21^ Fretwell et al.^28^ González-Montalvo et al.^29^ Harris et al.^30^ Landefeld et al.^22^ Olofsson^31^ Somme et al.^35^ Vidan et al.^39^	6,612	1.01 (0.81–1.27)	0.13 (.90)	11% (.33)
Costs (U.S. dollars standardized to 2000)^c^, ^d^	Asplund et al.^25^ Barnes et al.^41^ Counsell et al.^21^ Covinsky et al.^27^, ^b^ Stewart et al.^38^	4,287	–431.37 (–933.15–70.41)	1.68 (.09)	44% (.13)
Outlier removed	Asplund et al.^25^ Barnes et al.^41^ Counsell et al.^21^ Covinsky et al.^27^	4,226	–245.80 (–446.23 to –45.38)	2.40 (.02)	0% (.66)
Hospital readmissions	Asplund et al.^25^ Barnes et al.^41^ Counsell et al.^21^ Landefeld et al.^22^ Olofsson^31^	3,983	1.05 (0.92–1.18)	0.69 (.49)	0% (.55)

a Risk ratios (RRs) reported for all meta-analyses of all outcomes except cost and length of hospital stay, for which weighted mean difference (WMDs) are reported.

b Covinsky et al. 27 and Landefeld 1995^22^ refer to the same trial. Costs and length of hospital stay data extracted from Covinsky et al. 27

c Length of hospital stay and cost data from Collard and colleagues^26^ were excluded from meta-analyses; the reported standard errors were deemed erroneous because they contradicted their associated significance levels.^50^

d Costs were measured according to actual costs captured in hospital financial or accounting systems or charge data, which approximates costs of care using diagnostic information about each participant. When individuals were recruited into a study that covered a number of years, the cost year was presumed to be the middle year. When a year of recruitment was unavailable, the cost year was estimated to be 4 years before the publication date. Cost conversions performed June 22, 2012, using a Web-based cost converter endorsed by the Campbell and Cochrane Economics Methods Group.^20^

CI = confidence interval.

#### Length of Hospital Stay

Length of hospital stay was reported in 12 studies,^21^,^22^,^25^,^26^,^28^–^31^,^38^–^41^ with complete data in 11.^21^,^22^,^25^,^28^–^31^,^38^–^41^ Meta-analysis of these 11 studies showed that individuals receiving acute geriatric unit care experienced a significantly shorter length of hospital stay than those receiving usual care (WMD = −1.28, 95% CI = −2.33 to −0.22; *P* = .02). Significant statistical heterogeneity was observed between studies for this comparison. After removal of seven outlier studies^25^,^28^–^31^,^38^,^39^ during sensitivity analysis, the significant effect remained (WMD = −0.61, 95% CI = −1.16 to −0.05; *P* = .03), and statistical heterogeneity resolved (Table 1).

#### Discharge Destination

Nine studies reported whether participants were discharged home.^22^,^25^,^26^,^28^–^31^,^35^,^41^ Meta-analysis of these nine studies identified that individuals receiving acute geriatric unit care were 1.05 times more likely to be discharged home than those receiving usual care (RR = 1.05, 95% CI = 1.01–1.10; *P* = .01).

Six studies reported whether participants were discharged to a nursing home.^21^,^25^,^26^,^28^–^30^ Meta-analysis of these six studies identified no significant effect, although significant statistical heterogeneity was observed between studies for this comparison (Table 1). With the removal of three outlier studies^26^,^28^,^29^ that resolved the heterogeneity, a meta-analysis identified that individuals receiving acute geriatric unit care were significantly less likely than those receiving usual care to be discharged to a nursing home (RR = 0.82, 95% CI = 0.68–0.99; *P* = .04).

#### Mortality

Mortality was reported in 11 studies.^21^,^22^,^25^,^26^,^28^–^31^,^35^,^39^,^41^ Meta-analysis of these 11 studies identified no significant difference in mortality during hospital stay between individuals receiving acute geriatric and usual care (Table 1).

### Costs

Costs were reported in six studies,^21^,^25^–^27^,^38^,^41^ with complete data in five studies.^21^,^25^,^27^,^38^,^41^ Meta-analysis of these five studies showed that the costs of acute geriatric unit care were nonsignificantly less than the costs of usual care (WMD = −$431.37, 95% CI = −$933.15–$70.41; *P* = .09), although clinical heterogeneity was observed between studies for this comparison, as indicated by the minimal overlap of one study's CIs^38^ with those of the other studies (Appendix S3 of the electronic supplementary material). Heterogeneity was resolved with removal of one outlier study^38^ during sensitivity analysis; the results demonstrated that the costs of acute geriatric unit care were significantly less than those of usual care (WMD = −$245.80, 95% CI = −$446.23 to −$45.38; *P* = .02).

#### Hospital Readmissions

Two studies reported on hospital readmissions within 1 month of discharge,^21^,^31^ and three reported on hospital readmissions within 3 months of discharge.^22^,^25^,^41^ Meta-analysis of these five studies identified no significant difference in hospital readmissions within 1 or 3 months of discharge between individuals receiving acute geriatric unit care and those of individuals receiving usual care (Table 1).

#### Post Hoc Analyses

Post hoc subgroup meta-analyses were performed in the three studies that examined the effect of the full ACE model on the study outcomes. Results remained significant (length of hospital stay)^21^,^22^,^41^ or nonsignificant (functional decline between baseline hospital admission status and discharge, mortality, and hospital readmissions)^21^,^22^,^41^ or were inconclusive because of heterogeneity (discharge home)^22^,^41^ or no longer significant (functional decline between baseline 2-week prehospital admission status and discharge^21^,^22^,^41^ and costs^21^,^27^,^41^). The last may have been because of low power resulting in a Type I error.

## Discussion

This is the first combined systematic review and meta-analysis of acute geriatric unit care based on all or part of the ACE model components and the first to examine iatrogenic complications and functional decline between baseline hospital admission status and discharge. Results from meta-analyses demonstrate that acute geriatric unit care including one or more ACE components and introduced during the acute illness or injury phase has significant beneficial effects over usual care in reducing falls, delirium, functional decline between baseline 2-week prehospital admission status and discharge, length of hospital stay, discharge to a nursing home, and costs and in increasing discharges to home. In addition, a nonsignificant trend of finding fewer pressure ulcers was observed. Given the demographic and health characteristics of the average study participant, these findings are mainly applicable to octogenarians admitted through the emergency department with acute illnesses or injuries and other morbidities.

### Implications for Practice and Policy

The findings have relevance for clinicians, hospital administrators, policy-makers, and funders. By implementing all or part of the ACE model components during older adults' acute illness or injury phase, clinicians may anticipate small to moderate beneficial effects on the outcomes found to be significant in the meta-analyses. Although further research is needed, clinicians may also anticipate fewer pressure ulcers. Patient-centered care, frequent medical review, early rehabilitation, and early discharge planning were provided in more than half the studies and may represent the optimal ACE components for positive outcome achievement. Interdisciplinary team work was also a unique characteristic of acute geriatric unit care and may be important for clinicians to consider in their practice.

The findings are applicable to the care of acutely ill and injured older adults on medical, surgical, and medical–surgical units, and they address concerns about limited applicability and benefit of ACE to nonmedical patient groups and units.^42^ Older adults with acute injuries typically have comorbidities that precipitated the injury and complicate its management and therefore benefit from a function-focused prehabilitation approach.

Hospital administrators may anticipate cost savings of approximately $246 per hospital stay in U.S. dollars standardized to 2000 and more than a half-day shorter hospital stay than with usual care. Older adults account for 50% of Canadian^43^ and 45% of U.S.^44^ hospital expenditures. With projected increases in age demographics in both countries,^3^ this cost difference may represent a significant future source of financial saving to both healthcare systems. This finding addresses cost-ineffectiveness^12^ and cost-prohibitiveness^45^ barriers to adopting the ACE model.

By establishing ACE as the preferred model of care, policy-makers can play an influential role in its adoption and in the improvement of patient- and system-level outcomes. By changing reimbursement or charge rates and by establishing targets for cost and resource efficiency for older people's care, funders can create the external and substantive structural incentives needed to move ACE into the “mainstream of hospital care.”^46^

### Comparison with Previous Research

The findings of the current study are similar to those of an earlier meta-analysis of older adults with medical disorders^18^ that found that acute geriatric unit care, which may or may not have included the ACE components, had significant effects on preventing functional decline between baseline 2-week prehospital admission status and discharge, increasing discharges home, and reducing costs and nonsignificant effects on mortality and hospital readmissions. However, in contrast to the earlier meta-analysis, which found nonsignificant or inconclusive effects on length of hospital stay or discharges to a nursing home,^18^ the current meta-analysis identified significant reductions in length of hospital stay and discharges to a nursing home after acute geriatric unit care in which ACE components were provided in varying degrees. These findings concur with a prior narrative analysis comparing ACE with usual care units.^15^

This review included six new randomized^31^,^35^,^41^ and quasi-experimental^29^,^39^,^40^ trials, which resulted in larger sample sizes of many of the meta-analyses than in prior research. CIs were also more precise for most outcomes than were those of prior meta-analyses.^18^

### Strengths and Limitations of the Review

This review had little missing data because six study authors^21^,^27^,^29^,^35^,^39^,^41^ provided unpublished data, minimizing publication bias. The review included a small number of studies with limited information regarding study methods, which restricted the ability to draw conclusions regarding level of bias in several domains. Although randomization was used in most studies, six^21^,^25^,^26^,^35^,^39^,^40^ had postrandomization exclusions or did not report related information, which may have contributed to an overestimation of effect sizes. Sample sizes in the meta-analyses on iatrogenic complications were modest, which may have influenced the imprecision of the estimates.

Although this review included a diverse group of individuals admitted to medical, medical-surgical, or surgical units, heterogeneity was low in the majority of meta-analyses, supporting validity of the results. It was not possible to perform subgroup meta-analyses (medical vs surgical) because three studies did not report results separately for medical and surgical patients^26^,^28^,^38^ and because of the potential for bias with small and uneven distribution of groups.^20^

### Implications for Future Research

This review highlights the limited number of studies examining the effectiveness of acute geriatric unit care based on all or part of the ACE model components on outcomes of importance to older adults and service providers, specifically iatrogenic complications, costs, and hospital readmissions. With increasing concerns about safe and fiscally responsible care that does not result in hospital readmissions,^47^ future research should examine the effectiveness of acute geriatric unit care on these outcomes.

Future research should explore the effectiveness of the ACE components with surgical patients. As ACE continues to be adopted and tested in the care of older surgical patients, future researchers may conduct subgroup analyses to compare its effectiveness in medical patients with its effectiveness in surgical patients.

Most studies restricted entry to individuals admitted through the emergency department. Given the importance of community services,^48^ future trials should include older adults admitted to the hospital through avenues other than the emergency department. Future trials should also provide more-detailed descriptions of the methods used to facilitate assessment of the risk of bias and interpretation of results.

A prior meta-analysis^18^ examining the effectiveness of admission to acute geriatric units excluded studies that limited admission to acutely injured individuals but included studies with mixed samples of acutely ill and injured individuals. Inclusion of both types of studies in the current analysis did not lead to any more heterogeneity than previously reported, supporting the inclusion of acutely ill and injured older individuals in future meta-analyses.

Last, this review illustrates that few trials have examined the effectiveness of the full ACE model. Future updates of this review may enable new studies that explore the full ACE model to be incorporated into these subgroup meta-analyses, which will help to more accurately determine the effectiveness of the full ACE model.

## SUPPORTING INFORMATION

**Appendix S1.** Search strategy for MEDLINE (OVID).

**Appendix S2.** Descriptive characteristics of studies included in the systematic review and meta-analysis.

**Appendix S3.** Forest plots for all meta-analysis, including sensitivity analyses.
