# Supplementary material for: Effectiveness of Acute Geriatric Unit Care Using Acute Care for Elders Components: A Systematic Review and Meta-Analysis
Source: J Am Geriatr Soc. 2012 Nov 23;60(12):2237–45. doi: 10.1111/jgs.12028 (PMC3557720; doi:10.1111/jgs.12028)

**Supplemental files for Online Only**

**Appendix S1.** Search Strategy for MEDLINE(OVID)

**Limits applied:**

*Published Date:* yr="1985 -Current"

*Age Groups:* "all aged (65 and over)"

*Languages:*  English or French

*Organism:* humans

*Study Types:* case reports or clinical trial, all or clinical trial or comparative study or controlled clinical trial or meta analysis or multicenter study or randomized controlled trial or "review"

**A1 – Subject Heading Searches**

**Population:**

aged/ or "aged, 80 and over"/ or frail elderly/ or Geriatrics/ or veterans/

**Assessment and Aftercare:**

Geriatric Assessment/ or Health Services for the Aged/ or aftercare/ or comprehensive health care/ or patient care planning/ or advance care planning/ or progressive patient care/ or patient care team/ or disability evaluation/ or patient education as Topic/ or Case Management/ or Critical Pathways/ or Program Evaluation/

OR Health Status/ or health/ [limit to yr="1985 - 1989"]

**Hospital Units:**

Hospital Units/ or Intensive Care Units/ or Emergency Medical Services/ or emergency service, hospital/ or trauma centers/ or hospitals/ or intensive care units/ or coronary care units/ or recovery room/ or respiratory care units/ or operating rooms/ or rehabilitation centers/ or General Surgery/ or medicine/ or emergency medicine/ or internal medicine/ or Hospitals, Veterans/ or Acute disease/ or Psychiatric Department, Hospital/ or Hospitals, Psychiatric/

**Acute Conditions:**

exp "bacterial infections and mycoses"/ or exp virus diseases/ or exp parasitic diseases/ or exp neoplasms/ or exp musculoskeletal diseases/ or exp digestive system diseases/ or exp stomatognathic diseases/ or exp respiratory tract diseases/ or exp otorhinolaryngologic diseases/ or exp nervous system diseases/ or exp eye diseases/ or exp male urogenital diseases/ or exp "female urogenital diseases and pregnancy complications"/ or exp cardiovascular diseases/ or exp "hemic and lymphatic diseases"/ or exp "congenital, hereditary, and neonatal diseases and abnormalities"/ or exp "skin and connective tissue diseases"/ or exp "nutritional and metabolic diseases"/ or exp endocrine system diseases/ or exp immune system diseases/ or exp "wounds and injuries"/ or exp "pathological conditions, signs and symptoms"/ or acute disease/ or exp "psychiatry and psychology (non mesh)"/ or exp Accidents/

**Complications:**

postoperative complications/ or pain/ or pain, intractable/ or pain, postoperative/ or shock, surgical/ or surgical wound infection/ or venous thrombosis/ or Accidental Falls/ or Immobilization/ or Restraint, Physical/ or hip fractures/ or femoral neck fractures/ or osteoporotic fractures/ or Cognition Disorders/ or Delirium/ or confusion/ or Cognition/ or Delirium, Dementia, Amnestic, Cognitive Disorders/ or Depression/ or Affect/ or polypharmacy/ or Drug Monitoring/ or mood disorders/ or skin ulcer/ or leg ulcer/ or pressure ulcer/ or Ulcer/ or malnutrition/ or protein deficiency/ or protein-energy malnutrition/ or Nutritional Status/ or Elder Nutritional Physiological Phenomena/ or dehydration/ or starvation/ or wasting syndrome/ or Fecal Incontinence/ or Urinary Incontinence/ or Urinary Catheterization/ or Iatrogenic Disease/ or Cross Infection/ or catheter-related infections/ or pneumonia/ or soft tissue infections/ or wound infection/ or Urinary Tract Infections/ or Catheters, Indwelling/ or sleep disorders/ or sleep deprivation/ or sleep disorders, circadian rhythm/ or sleep disorders, intrinsic/ or "disorders of excessive somnolence"/ or "sleep initiation and maintenance disorders"/ or Dizziness/ or syncope/ or syncope, vasovagal/ or vertigo/ or Mobility Limitation/ or Hospital Mortality/ or Mortality/ or Vision, Low/ or Hearing Loss, Bilateral/ or Hearing Loss, Functional/ or Hearing Loss/

**Cost and Cost Analysis:**

"Costs and Cost Analysis"/ or Cost allocation/ or Cost-benefit analysis/ or Cost control/ or Cost savings/ or Health care costs/ or Hospital costs/

**Rehabilitation:**

Rehabilitation/ or "activities of daily living"/ or early ambulation/ or exp exercise therapy/ or occupational therapy/ or treatment outcome/ or physical therapy modalities/ or exercise movement techniques/ or breathing exercises/ or exercise therapy/ or motion therapy, continuous passive/ or muscle stretching exercises/ or resistance training/ or music therapy/ or recovery of function/ or exercise/ or Self care/ or medication therapy management/ or Nutrition therapy/ or diet therapy/ or nutritional support/ or enteral nutrition/ or Parenteral Nutrition/ or nutrition assessment/ or Nutritional Status/ or Fluid therapy/ or Accident prevention/ or safety/ or primary prevention/ or secondary prevention/ or tertiary prevention/ or Safety Management/ or Patient care/ or aftercare/ or critical care/ or intensive care/ or perioperative care/ or intraoperative care/ or postoperative care/ or preoperative care/ or night care/ or patient positioning/ or perioperative nursing/ or emergency nursing/ or geriatric nursing/ or operating room nursing/ or postanesthesia nursing/ or rehabilitation nursing/ or "moving and lifting patients"/ or patient positioning/ or perioperative period/ or intraoperative period/ or postoperative period/ or preoperative period/ or Psychiatric Nursing/ or "Physical Therapy (Specialty)"/ or Physical Therapy Modalities/ or Occupational Therapy/ or Physical Medicine/ or Primary Nursing/ or Nursing Assessment/ or Nursing, Team/ or Nursing Process/ or Patient Care Planning/ or Social Work, Psychiatric/ or Social Work/ or Pharmacists/ or physicians/ or general practitioners/ or physicians, family/ or quality of health care/ or risk factors/ or Caregivers/ or Clinical Protocols/ or Therapeutics/ or Environment Design/ or "Interior Design and Furnishings"/ or Toilet Facilities/ or Self-Help Devices/ or Wheelchairs/ or Hearing Aids/ or Protective Devices/ or "Continuity of Patient Care"/ or exp Bandages/

OR Primary Health Care/ [limit to yr="1985 - 1990"]

**Hospitalization and Discharge:**

hospitalization/ or "length of stay"/ or patient admission/ or patient discharge/ or patient readmission/ or patient transfer/ or patient care management/ or patient-centered care/ or institutionalization/

**Delivery of Healthcare:**

"delivery of health care"/ or "delivery of health care, integrated"/ or "quality of health care"/ or "outcome and process assessment (health care)"/ or "outcome assessment (health care)"/ or treatment outcome/ or "process assessment (health care)"/ or program evaluation/ or quality assurance, health care/ or "patient acceptance of health care"/ or needs assessment/ or health promotion/ or "Health Services Needs and Demand"/ or Preventive Health Services/ or Goals/ or Social Adjustment/

**A2 – Keyword Searches**

**Population:**

(aged or frail* or elder* or Geriatric* or (old* adj2 (person* or people)) or veteran* or octogenarian* or nonagenarian* or centenarian* or gerontol*).mp.

**Assessment, Aftercare, Programs:**

((aged or frail* or elder* or Geriatri* or (old* adj2 (person* or people or patient* or client*)) or veteran* or octogenarian* or nonagenarian* or centenarian* or gerontol* or patient*) adj3 (Assess* or evaluat* or manag* or apprais* or function* or Health Service* or aftercare or after-care or acute care or acute-care or comprehensive* or (patient* adj3 plan*) or (advance* adj3 plan*) or (progressive adj3 care) or patient care team or disability evaluation or patient* education or (Case adj3 Manag*) or manage* care program* or Critical Pathway* or Program* Evaluation or Health Education)).mp.

OR (((consultative or comprehensive or evaluat*) adj3 geriatric* assessment) or (geriatric evaluati* adj3 management unit*) or stroke unit* or stroke team* or quality improvement program* or (geriatric* patient* adj3 care) or geriatric* inpatient* service* or early support* discharge* or intens* motor train* or comprehensive stroke unit* or (improve* adj3 program*) or orthogeriatric unit* or (hospital adj2 home) or hospital-at-home or multidisciplinary rehabilitat* or Motor Assessment Scale* or MAS or (Inpatient rehabilitat* adj3 unit*) or discharge destination* or (co?ordinated adj3 rehabilitat*) or (community?based adj3 rehabilitat*) or Early?supported discharge rehabilitat* or (reduce* length adj2 hospital*) or Post?discharge rehabilitat* or (transfer* adj3 (patient* adj3 hospital*)) or (transfer* adj3 (patient* adj3 communit*)) or General practitioner?oriented or post?stroke rehabilitat* or hospital rehabilitat* or community rehabilitat* or (geriatric* evaluat* adj3 treat* unit*) or transition* care bridge or vulnerable elder* survey or geriatric* care program* or hospital elder life program* or (Acute Care adj3 Vulnerable Elder*) or (Nurse* Improv* Care adj3 Health?System Elder*) or (Hospital Elder Life Program) or (Nurses Improving Care to Health System Elders) OR (Program of All-Inclusive Care for the Elderly) or (Yale Geriatric Care Program) ).mp.

**OR** ((Medicat* adj3 review*) or (rehabilitat* adj3 model*) or (inpatient* adj3 rehabilit*) or (dementia adj3 service*) or (care adj3 transition*) or (interdisciplinary adj3 transition*) or (nutrition* adj3 supplement*) or (pressure adj3 relie*) or ((elder* or geriatric* or senior*) adj3 (interven* or consult* or Prevent* or evaluat* or manag* or unit* or nursing or program* or Post-Acute Care* or acute care or section* or care unit* or care-unit* or Treatment* or Integrated service* or resource* or assess*)) or (multicomponent hospital-based intervention* or Psychogeriatric) or (Biopsychosocial adj3 (Evaluat* or Treatment* or Program*)) or Hospital in the Home* or translating research into practice* or ((Geriatric* Resource* adj2 (Assess* adj3 Care)) and Elders*) or ((Reduc* adj3 Fall*) and Elder*) or interdisciplinary comprehensive geriatric* assessment* or Senior care unit*).mp.

**OR** (Mobile geriatric* or geriatric* team* or (geriatric* and float* and interdisciplinary and transition and team*) or comprehensive geriatric* intervention* or acute geriatric* unit* or geriatric intervene* or (acute care adj3 elder*) or geriatric consult* team* or (integrated care adj3 elder*) or geriatric* rehabilitation or (multidisciplinary adj3 care)).mp.

**Hospital Units:**

(acute*adj2 care* or emerg* or ((Emergenc* or hospital* or veteran* or trauma* or health* or care or surveillan* or coronary* or cardiac* or Intensive* or recover* or special* or geriatr* or elder* or aged* or operat* or respirat* or rehabilitat* or surg* or post-surg* or general* or medical* or inpatient* or in-patient*) adj3 (room* or department* or unit* or ward* or service* or care* or facilit*))).mp.

**Cost and Cost Analysis:**

((multi* adj3 (fact* or compo* or facet*)) or efficien* or reinforc* or cost-effect* or cost* or utilit* or economic* or nursing-led or interdisciplin* or multidisciplin* or pharmac* or inter-disciplin* or multi-disciplin* or social work* or social-work* or physic* therap* or physio-therap* or physical-therap* or occupational therap* or occupational-therap* or strateg* or best practic* or outcom* or quality of life or quality-of-life or adaptat* or success* or prepar* or method* or manag* or benefit* or plan* or coach* or innovat* or initiat* or influenc* or alternat* or promot* or integrat* or disseminat* or adopt* or occur* or prevail* or preval* or follow-up* or follow* up* or result* or servic* or restor* or implication* or detect* or enrich* or promot* or incident* or train* or collaborat* or measur* or rate* or rati* or control* or develop* or feasibl* or feasibil* or evaluat* or regimen* or reduc* or benefit* or interven* or design* or redesign* or re-design* or method* or inciden* or recover* or protect* or coordinat* or co-ordinat* or progres* or chang* or early or earlier or timing or time* or transition* or function* or Supplement* or Minimiz* or minimis* or design* or establish* or servic* or support* or supplement* or screen* or discharg* or compar* or advis* or advic* or teach* or taught or learn* or follow-up or follow* up).mp.

**Rehabilitation:**

(Rehabilitat* or activities of daily living or early ambulat* or (exercise* adj2 therap*) or occupational therap* or (treatment adj2 outcome*) or physical therap* or exercise* movement technique* or breathing exercise* or motion therap* or muscle stretch* or resistance train* or music therap* or recovery of function* or exercise* or Self care* or medication therap* or Nutrition therap* or diet therap* or nutritional support* or enteral nutrition* or Parenteral Nutrition* or nutrition assessment* or Nutritional Status* or Fluid therap* or Accident prevent* or safety or primary prevent* or secondary prevent* or tertiary prevent* or Patient care or aftercare or (critical adj3 (care or nurs* or period*)) or (intensive adj3 (care or nurs* or period*)) or (perioperative adj3 (care or nurs* or period*)) or (peri-operative adj3 (care or nurs* or period*)) or (intraoperative adj3 (care or nurs* or period*)) or (intra-operative adj3 (care or nurs* or period*)) or (post-operative adj3 (care or nurs* or period*)) or (postoperative adj3 (care or nurs* or period*)) or (pre-operative adj3 (care or nurs* or period*)) or (preoperative adj3 (care or nurs* or period*)) or night* care or night?care or (emergency adj3 nurs*) or (geriatric adj3 nurs*) or (operat* room* adj3 (nurs* or care)) or ((postanesthe* or post-anesthe* or postanaesthe* or post-anaesthe*) adj3 (nurs* or care)) or (patient adj3 (position* or mov* or lift*)) or Psychiatr* Nurs* or Physical Medicine or Primary Nurs* or ((Nurs* or interdisciplin* or multidisciplin* or inter-disciplin* or multi-disciplin*) adj3 (Team* or process* or plan* or assess* or process*)) or (Patient adj2 Plan*) or Social Work* or social-work* or Pharmac* or physician* or general practitioner* or Therapeutic* or (Environment* adj2 Design*) or Toilet* or ((Self-Help* or self help*) adj3 Device*) or Wheelchair* or wheel-chair* or Hearing Aid* or hearing-aid* or (Protect* adj3 Device*) or Bandage*).mp.

**OR** (multi-state model* or ((structure* adj3 education*) and model*) or multi-component or (multi* adj3 component*) or hospital-base* or (hospital adj3 base*) or (stretch* adj3 exercise*) or (physical* adj3 psychosocial function*) or (support adj3 surface*) or pressure ulcer* or multi-factor* or multifactor* or (exercise adj3 base*) or fast track* or fast-track* or vulnerable or (disabilit* adj2 prevent*) or (quality improve* adj3 intervention*) or hospital-based or post-acute or ((diet* or nutrition*) adj3 (supplement* or support*)) or fall-prevent* or fall* prevent* or vitamin or ((one-time or teach-to-goal or education*) adj3 (intervention or program)) or quality improve* or (reduce* adj3 pain) or recovery or (function* adj3 assess*) or (diet* adj3 assistant*) or physiotherap* or (discharge adj3 destination*) or (length* adj3 stay*) or complication* or (multi-state adj3 model*) or ((multi adj3 state) and model*) or trunk control test or risk assess* or nursing interven* or (restrain* adj3 reduc*) or supplementation or (hospital and discharge plan*) or (repetitive task adj3 train*) or music or ((improve* adj3 function*) and ability*) or malnutrition or mattress* or relax* or reinforc* or ((reduce* adj3 function*) and decline*) or (function* adj3 decline*)).mp.

**OR** (treat* or effect* or effic* or prevent* or enhanc* or model* or remodel* or motivati* or function* or reduc* or organiz* or improv* or outcom* or therap* or evaluat* or valu* or interven* or comprehen* or rehabilitat* or train* or exercis* or Transition* or Detect* or Examin* or characteri* or target* or impact* or identif* or support* or compar* or supplement* or progress* or comprehen* or implement*).mp.

**OR** (((massage or physi* or occupation*) adj3 therap*) or postoperat* or discharg* plan*).mp.

**Hospitalization and Discharge:**

(discharg* or admit* or admis* or readmit* or readmis* or hospitaliz* or process* or fall-prevent* or fall* prevent* or approach* or multidisciplin* or multi-disciplin* or interdisciplin* or inter-disciplin* or benefit* or physical function* or recover* or motivation* or assist* or factor* or approach* or delay* or (support* adj3 discharg*) or post-discharg* or post discharg* or mobiliz* or mobilis* or program* or decreas* or supplement* or strateg* or optim* or assess* or multifactor* or multicompo* or multifacet* or multi-factor* or multi-compo* or multi-facet*).mp.

**Delivery of Healthcare:**

((((health* adj 2 care) or health-care or healthcare) adj3 (deliver* or distribut* or system* or activit* or non-clinical* or non clinical* or communit* or quality or standard* or excellence or outcome* or out-come or process*)) or (outcome* adj3 (assess* or stud* or measure* or process* or treat*))).mp.

| **Appendix S2**. Descriptive characteristics of studies included in the systematic review and meta-analysis. | | | | | | | |
| --- | --- | --- | --- | --- | --- | --- | --- |
| Table S1. Characteristics of Included Studies | | | | | | | |
|  | | Comparison Groups | | Comparison Units | |  |  |
| Study,^a^  Design, and Setting | Target Population and Study Enrolment Time | Acute Care Geriatric  Intervention | Usual Care | Acute Care Geriatric Intervention | Usual Care^b^ | Time of Assessment | Outcomes^c^ |
| Asplund et al.^[25](#_ENREF_25)^  RCT.  Sweden. | Aged > 70 years admitted with acute medical illnesses to emergency department not requiring admission to a specialized, intensive care, coronary care, stroke, or renal unit.  In emergency department. | n = 190; mean age = 81, 58% female, 16% living in institutions, 47% cognitively impaired on admission.  Admitting diagnostic category or symptom: chest pain (21%), dyspnea (18%), nausea/vomiting (11%), vertigo (11%), other pain (8%).  Co-morbidities:  angina (25%), MI (24%), diabetes (17%), stroke (16%), history of cardiac failure (13%), dementia (6%). | n = 223; mean age = 81, 63% female, 16% living in institutions, 52% cognitively impaired on admission.  Admitting diagnostic category or symptom: chest pain (25%), dyspnea (18%), other pain (13%), nausea/vomiting (11%), vertigo (11%).  Co-morbidities:  angina (27%), diabetes (23%), stroke (22%), MI (17%), history of cardiac failure (15%), dementia (5%). | 1 acute geriatric ward.  Patient-centered care.  Frequent medical review.  Early rehabilitation.  Early discharge planning.  Interdisciplinary team directed.  Team: geriatrician, internist, nurses, dietician, occupational therapist, physiotherapist.  Team education prior to trial: interdisciplinary team working principles, geriatric care, and ethics issues. | General medical wards.  Discharge planning initiated shortly before discharge.  Not interdisciplinary team directed.  Team: physician, internist, nurses.  No geriatrician. No routine service from dietician, social worker, occupational therapist, or physiotherapist (but available). | Hospital admission, discharge, and 3 months post discharge. | Delirium, length of hospital stay, discharge destination (home, geriatric rehabilitation ward, other hospital ward, nursing home, sheltered living, and preadmission residence) mortality, cost of hospital stay, hospital readmissions, cognitive functioning, psychological well-being, activities of daily living function, and poor global outcome. |
| Barnes et al.[^41^](#_ENREF_41)  RCT  USA | Aged > 70 years admitted to emergency department, not requiring admission to a specialty unit (e.g. intensive care or other subspecialty unit). Excluded patient with expected length of stay < 2 days and elective admissions.  Upon admission. | n = 858; mean age = 80.6, 67% female, 16% living in boarding home, assisted living, or nursing home on admission.  Admitting diagnostic category or symptom: neurologic (11%); cardiovascular (14%); pneumonia/infection (12%); pulmonary (21%); hematologic (2%); gastrointestinal (20%); or metabolic (18%). Comorbidities: congestive heart failure (28%), cancer (7%), chronic lung disease (17%), history of myocardial infarction (15%), cerebrovascular disease (17%), dementia (18%); peripheral vascular disease (5%). Mean Charlson comorbidity score = 1.9. | n = 774; mean age = 80.6, 67% female, 16% living in boarding home, assisted living, or nursing home on admission.  Admitting diagnostic category or symptom: neurologic (10%); cardiovascular (14%); pneumonia/infection (14%); pulmonary (22%); hematologic (3%); gastrointestinal (20%); or metabolic (17%). Comorbidities: congestive heart failure (26%), cancer (7%), chronic lung disease (17%), history of myocardial infarction (13%), cerebrovascular disease (16%), dementia (19%); peripheral vascular disease (8%). Mean Charlson comorbidity score = 1.8. | 1 ACE unit.  Patient-centered care.  Frequent medical review.  Early rehabilitation.  Early discharge planning.  Prepared environment.  Team: nurses, geriatric clinical nurse specialist, geriatrician, physicians, social workers, physiotherapists.  Team meetings: daily. | 1 general medical unit.  Not team based approach to providing care.  Team members: nurses and physicians. Other members not described.  One year after trial initiated, usual care unit relocated to new building with similar prepared environment to ACE unit and several ACE  protocols implemented hospital-wide. | Hospital admission, discharge, and 3 months after discharge. | Functional decline at discharge from baseline 2-week pre-hospital and hospital admission status, length of hospital stay, discharge destination (home), mortality, cost of hospital stay, hospital readmissions , instrumental activities of daily living change, mobility change. |
| Collard et al.[^26^](#_ENREF_26)  RCT.  Two community hospitals, USA. | Aged > 65 years and expected length of stay > 48 hours.  Patients could be transferred to the intervention unit from intensive care unit (ICU).  Upon admission. | n = 218; mean age = 78.4, 56.4% female, 10% admitted from nursing home. Admitting diagnostic category or symptom: cerebrovascular (7%), neurological (7%), fracture (6%), cardiac (5%), bowel/intestinal (4%),  metastatic malignancy (4%), respiratory (12%),  and other (45%). | n = 477; mean age = 77.8, 42.8% female, 8% admitted from nursing home. Admitting diagnostic category or symptom: respiratory (13%), cardiac (11%), cerebrovascular (7%), fracture (7%), bowel/intestinal (4%), neurological (4%), metastatic malignancy (4%), and other (50%). | 2 geriatric special care units at 2 hospitals.  Patient-centered care.  Early discharge planning.  Team: nurses, occupational therapists, physicians, medical director, physiotherapists, and social workers.  Team meetings: twice weekly. | Traditional medical-surgical units at 2 hospitals.  Not described. | Hospital admission, discharge, 3 weeks post discharge, and 6 months post discharge. | Falls, skin breakdown, length of hospital stay, discharge destination (routine/home, home with home health care, acute hospital, nursing home, home health care, other), mortality, cost of hospital stay, other hospital complications (pneumonia, confusion, infection). |
| Counsell et al.[^21^](#_ENREF_21)  RCT.  Community hospital, USA. | Community dwellers aged > 70 years admitted to emergency department not requiring admission to specialty unit (e.g. ICU, coronary care unit (CCU), telemetry, and oncology).  Excluded transfers from nursing facilities or other hospitals, length of stay < 2 days or previously enrolled in study.  Upon admission. | n = 767; mean age = 80 years, 60% female, no nursing home admissions. Admitting diagnostic category or symptom: dyspnea/pulmonary problem (25%); altered mental status or neurological abnormality (19%); gastrointestinal problem (19%); congestive heart failure, chest pain, or cardiac problem (11%); fever, pneumonia, or infection (14%); diabetes mellitus, failure to thrive, other problem (12%). Comorbidities: congestive heart failure (30%), chronic lung disease (27%), cardiovascular disease (21%), dementia (16%). Mean Charlson comorbidity score = 2.5. | n = 764; mean age = 79, 61% female, no nursing home admissions.  Admitting diagnostic category or symptom: dyspnea/pulmonary problem (23%); altered mental status or neurological abnormality (21%); gastrointestinal problem (19%); congestive heart failure, chest pain, or cardiac problem (14%); fever, pneumonia, or infection (12%); diabetes mellitus, failure to thrive, or other problem (12%).  Comorbidities: congestive heart failure (28%), cardiovascular disease (22%), chronic lung disease (21%), dementia (18%). Mean Charlson comorbidity score = 2.5. | 1 acute care for elders (ACE) unit.  Patient-centered care.  Frequent medical review.  Early rehabilitation.  Early discharge planning.  Prepared environment.  Team: nurses, geriatric clinical nursing specialist, medical director, geriatrician, attending and resident physicians, social worker, dietitian, physical and occupational therapists, and home care coordinator.  Team education prior to trial: geriatric care.  Team meetings: daily. | Medical units.  Not interdisciplinary team directed.  Not functionally focused.  Team: not described other than similar nurse to patient ratio as ACE unit. | Hospital admission, discharge, and 1, 3, 6, and 12 months after discharge. | Functional decline at discharge from baseline 2-week pre-hospital and hospital admission status, length of hospital stay, discharge destination (nursing home), mortality, hospital charges, hospital readmission, process of care, patient and staff satisfaction. |
| Fretwell et al.[^28^](#_ENREF_28) Owens et al.[^34^](#_ENREF_34)  RCT.  USA. | Aged > 75 years, not on protocol treatment or requiring intensive or coronary care. Patients ready for transfer from ICU and CCU eligible.  Within 24 hours of admission to intervention or usual care unit. | n = 221; mean age = 83, 72% female, 71% from nursing home, 85% cognitively impaired on admission. Admitting diagnostic category or symptom: other than acute episode, not specified. | n = 215; mean age = 83, 72% female, 75% from nursing home, 83% cognitively impaired on admission.  Admitting diagnostic category or symptom: other than acute episode, not specified. | 1 senior care unit, described as a medical ward.  Patient-centered care.  Frequent medical review.  Early rehabilitation.  Team: nursing coordinator, dietitian, pharmacist, physician (specialized in geriatrics), physiotherapist, and social worker.  Team meetings: 3 times per week. | General medical surgical units.  Standard medical care of the hospital.  Team: not identified. | Hospital admission, discharge, 6 weeks after admission, 3 months post discharge, 6 months post discharge, and 2 years. | Functional decline in at 6 weeks post-discharge from baseline hospital admission status, length of stay, discharge destination (home and nursing home), mortality, hospital charges exceeding diagnosis related group reimbursement, cognitive functioning, and depression. |
| González-Montalvo et al.^[29](#_ENREF_29)^  Quasi-experimental randomized intervention study. Spain. | Aged > 65 years admitted to emergency department with osteoporotic hip fracture.  Upon admission to units. | n = 123; mean age = 85, 83% female, 52% history of cognitive impairment, 38% admitted from nursing home.  Admitting diagnostic category or symptom: osteoporotic hip fracture. | n = 101; mean age = 87, 83% female, 54% history of cognitive impairment, 33% admitted from nursing home.  Admitting diagnostic category or symptom: osteoporotic hip fracture. | 1 acute ortho-geriatric unit.  Patient-centered care.  Team: nurses, physiotherapist, geriatrician, and orthopedic surgeon.  Team meetings:  orthopedic surgeon and geriatrician met daily; orthopedic surgeon, geriatrician, physicians, nurses, and physiotherapist met weekly. | 1 orthopedic unit.  No geriatric assessment. No interdisciplinary weekly team rounds. No joint daily management by orthopedic surgeon and geriatrician.  Team: nurses, geriatrician (consulting basis only), orthopedic surgeon, and physiotherapist. | Hospital admission and discharge. | Length of hospital stay, discharge destination (own home, nursing home, long-term care units), mortality, and ability to walk with and without help. |
| Harris et al.[^30^](#_ENREF_30) Australia.  RCT. | Aged > 70 years admitted to emergency department. Excluded patients admitted to the hospital within past 7 years and nursing home residents.  In emergency department. | n = 97; mean age = 78.3, 35% female, no nursing home admissions. Admitting diagnostic category or symptom: unspecified acute illnesses. Comorbidities: cardiovascular (50%), cerebrovascular (13%), respiratory system (10%), mental disorder (7%), neoplasm (5%), ill-defined conditions (9%), endocrine (3%), nervous system (3%). | n =170; mean age = 78.3, 41% female, no nursing home admissions. Admitting diagnostic category or symptom: unspecified acute illnesses. Comorbidities: cardiovascular (47%), cerebrovascular (14%), respiratory system (17%), mental disorder (4%), neoplasm (4%), ill-defined conditions (25%), endocrine (3%), nervous system (2%). | 1 geriatric assessment unit (GAU).  Patient-centered care.  Discharge planning (not described as early).  Team: physician, geriatrician, nurses, clinical nursing specialist, occupational and physical therapist, and social worker. | 2 general medical units.  Discharge planning (not described as early).  Team: nurses and physician with access to allied health professionals but less dedicated time than on GAU. | Hospital admission and discharge. | Length of hospital stay, discharge destination (home and nursing home/hostel), and mortality. |
| Landefeld et al.[^22^](#_ENREF_22)  Covinsky et al.[^27^](#_ENREF_27)  RCT.  USA. | Aged > 70 years admitted to emergency department, not requiring admission to a specialty unit (e.g. intensive care, cardiology, telemetry, or oncology).  Upon admission. | n = 327; mean age = 80.2, 68% female, 3% living in institutions on admission.  Admitting diagnostic category or symptom: acute dyspnea or other pulmonary problem (19%); gastrointestinal (19%); congestive heart failure, chest pain or other cardiac problem (18%); fever, pneumonia, or other infection (18%); diabetes mellitus, failure to thrive, or other problem (14%); altered mental status or other neurologic abnormality (11%). Comorbidities: congestive heart failure (26%), cancer (23%), chronic lung disease (22%), history of myocardial infarction (17%), cerebrovascular disease (12%), dementia (10%). Mean Charlson comorbidity score = 2.3. | n = 324; mean age = 80.1, 65% female, 8% living in institutions on admission.  Admitting diagnostic category or symptom: fever, pneumonia, or other infection (20%); acute dyspnea or other pulmonary problem (20%); gastrointestinal (19%); diabetes mellitus, failure to thrive, or other problem (18%); congestive heart failure, chest pain or other cardiac problem (15%); altered mental status or other neurologic abnormality (13%).  Comorbidities: congestive heart failure (23%), cancer (21%), chronic lung disease (20%), history of myocardial infarction (21%), cerebrovascular disease (18%), dementia (13%).  Mean Charlson comorbidity score = 2.3. | 1 ACE unit.  Patient-centered care.  Frequent medical review.  Early rehabilitation.  Early discharge planning.  Prepared environment.  Team: nursing, dietician, resident, attending physicians, physiotherapist, occupational therapist, social worker. Increased hours provided for medical and nursing directors, social worker, physiotherapist, occupational therapist, and dietitian, which totaled less than 1 full-time person per year.  Team education prior to trial: geriatric care.  Team meetings: daily. | 1 general medical unit.  Services provided by physicians and nurses in other acute medical units.  Not interdisciplinary team directed.  Not functionally focused.  Team members: nurses, dietician,  resident, attending physicians,  physiotherapist, and social worker. | Hospital admission, discharge, and 3 months after discharge. | Functional decline at discharge from baseline 2-week pre-hospital and hospital admission status, length of hospital stay, discharge destination (home, long-term care institution (i.e., skilled nursing facility, rehabilitation hospitals, and other institutions providing assistance with ADL) and preadmission residence), mortality, cost of hospital stay,[^27^](#_ENREF_27) hospital charges,[^22^](#_ENREF_22) hospital readmissions, cognitive function, residence in a long-term care facility. |
| Olofsson^[31](#_ENREF_31)^  Lundstrom et al.[^33^](#_ENREF_33)  Olofsson et al.[^32^](#_ENREF_32)  Stenvall et al.[^36^](#_ENREF_36),[^37^](#_ENREF_37)  Sweden. | RCT.  Aged > 70 years, admitted to emergency department with hip fracture not sustained in hospital. Excluded patients with rheumatoid arthritis, severe hip osteoarthritis, severe renal failure, pathological fracture, and/or bedridden prior to fracture. | n = 102; mean age = 82, 74% female, 65% living independently.  Admitting diagnostic category or symptom: hip fracture.  Co-morbidities: cardiovascular diseases (56%), depression (32%), dementia (28%), stroke (28%), diabetes (23%), prior hip fracture (16%), cancer (15%). | n = 97; mean age = 82, 74% female, 62% living independently.  Admitting diagnostic category or symptom: hip fracture.  Co-morbidities: cardiovascular diseases (57%), depression (47%), dementia (37%), stroke (22%), diabetes (18%), cancer (15%), prior hip fracture (15%). | 1 geriatric ward.  Patient-centered care.  Frequent medical review.  Early rehabilitation.  Team education: geriatric care, rehabilitation, teamwork.  Team: geriatrician, orthopedic surgeon, nurses, geriatrician, occupational therapist, physiotherapist, social worker, and dietician.  Team meetings: twice a week. | 1 orthopedic ward.  Conventional post-operative care in an orthopedic ward.  Usual post-operative care routines which did not consistently include individualized care planning, early removal of catheters, prevention of complications,  interdisciplinary teamwork, active prevention, detection and treatment of delirium, or assessment by occupational therapist, physiotherapist, or geriatrician. No staff education.  Team: nurses, occupational therapist (fewer hours than geriatric ward), physiotherapist, orthopedic surgeon.  Other team members available only on a consulting basis.  No dietician. | Hospital admission, pre-operatively, post-operatively on days 1,2,3,4,5,6,7, discharge, 4 and 12 months after post-operative day 7. | Delirium, hospital complications (falls, pressure ulcer incidence, urinary tract infections, sleep disturbances, nutritional problems), functional decline at discharge from baseline 2-week pre-hospital admission status, length of hospital stay, discharge destination (pre-admission residence), mortality, hospital readmission within 2 months, and mental status. |
| Somme et al.[^35^](#_ENREF_35)  RCT.  France. | RCT. Aged > 74 years scheduled for transfer from medical ICU and cognitively able to provide informed consent. Excluded patients who had been transferred to ICU from an acute ward, and/or required cardiac surgery, neurosurgery, and/or invasive cardiac examinations.  In ICU. | n = 24; mean age = 81, 58% female, 8% living in institution.  Admitting diagnostic category or symptom (top 3 reported): acute respiratory failure (38%), congestive heart failure (38%), and sepsis (13%). | n = 21; mean age = 81, 52% female, 14% living in institution.  Admitting diagnostic category or symptom (top 3 reported): acute respiratory failure (38%), congestive heart failure (38%), and sepsis (13%). | 1 post-ICU ACE unit.  Patient-centered care.  Frequent medical review.  Early discharge planning.  Prepared  environment.  Team: nurses, dietician, physician, internist, physiotherapist, social worker, dietician, and neuropsychologist.  Team meetings: daily (physicians, chief nurse, and social worker) and weekly (all team members). | Post ICU standard wards (medical unit inferred from admitting diagnoses).  Similar nursing staff levels as ACE unit with access to an occupational therapist on a consulting basis. | Hospital/ICU admission, discharge from ICU, admission to post ICU unit/wards, hospital discharge, 7 days after hospital discharge. | Discharge destination (home and undefined institutions), mortality, and global physical autonomy. |
| Stewart et al.[^38^](#_ENREF_38)  Quasi-experimental controlled trial.  Community teaching hospital, USA. | Age > 75 years admitted under medical, general surgical, and orthopaedic services with diagnoses that met Medicare guidelines for acute care. Patients aged 75 to 84 years old required to meet frailty criteria. Patients with comorbidities and other frailty criteria given priority for selection. Excluded patients who were not fully independent or terminally ill. Within 72 hours of admission. | n = 34; mean age = 86, approximately 20% from nursing homes and other sources not identified as home.  Admitting diagnostic category or symptom: medical (80%), general surgical (15%), orthopaedic (5%).  Mean number of co-morbidities = 7. | n = 27; mean age = 82, approximately 20% from nursing homes and other sources not identified as home.  Admitting diagnostic category or symptom: unspecified medical and surgical (similar proportion of surgical patients as acute geriatric unit). Mean number of co-morbidities = 5. | 1 acute GEM unit.  Patient-centered care.  Team: registered nurses, licensed practical nurses, nursing assistants, social worker, and physician assistant with supervision from a geriatrician. | 1 medical-surgical unit.  Not described. | Hospital admission and at discharge. | Length of stay and cost of hospital stay. |
| Vidan et al.[^39^](#_ENREF_39)  Quasi-experimental controlled trial.  University hospital, Spain. | Aged > 70 years with no delirium on admission but > 1 delirium risk factors.  Within 24 hours of admission to unit. | n = 170; mean age = 86, 62% female, 63% cognitively impaired on admission, 44% high risk of delirium, 23% not living at home on admission. Admitting diagnostic category or symptom: heart failure (22%) and infectious diseases including respiratory infections, exacerbated chronic obstructive pulmonary disease, urinary tract infection (43%). Mean number of comorbidities = 2.6. | n= 372; mean age = 82, 53% female, 55% cognitively impaired on admission, 29% high risk of delirium, 14% not living at home on admission. Admitting diagnostic category or symptom: heart failure (21%) and infectious diseases including respiratory infections, exacerbated chronic obstructive pulmonary disease, urinary tract infection (43%). Mean number of comorbidities = 2.7. | 1 geriatric unit.  Patient-centered care.  Frequent medical review.  Prepared environment.  Team: nurses, geriatrician, residents, specialist geriatric nurse.  Team meetings: daily.  Team education prior to trial: geriatric care. | 2 internal medicine wards; standard care.  Team: nurses, internists. Dietician, rehabilitation team, and social worker available by consultation. | Hospital admission and discharge. | Falls, delirium, functional decline at discharge from baseline 2-week pre-hospital admission status, length of hospital stay, mortality, bed rest > 48 hours, and daily walking. |
| Zelada et al.^[40](#_ENREF_40)^  Quasi-experimental controlled trial. Peruvian Airforce Teaching Hospital, Peru. | Aged > 65 years admitted from emergency department with acute medical pathology. Excluded patients with pre-admission dependency in all basic ADLs, intubated, with severe dementia and/or aphasia, terminal cancer, expected to be discharged in < 24 hours, admitted for intensive care or specific procedures (e.g. cardiac catheterization, colonoscopies), transferred from other services, and referred to geriatric services.  Within 48 hours of admission. | n = 68; mean age = 79.6, 61.8% female. Admitting diagnostic category or symptom: respiratory (27%), other medical pathology (27%), gastrointestinal (18%), cardiovascular (9%).  Mean Charlson comorbidity score = 3.6. | n = 75; mean age = 76.1, 56% female. Admitting diagnostic category or symptom: cardiovascular (25%), respiratory (23%), other medical pathology (23%), gastrointestinal (13%). Mean Charlson comorbidity score = 3.1. | 1 geriatric care unit. Patient-centered care.  Frequent medical review.  Early rehabilitation.  Early discharge planning.  Team: nurses, geriatric physician, resident, social worker, physiotherapist, and occupational therapist.  Team meetings: weekly. | 1 acute care unit (medical unit inferred from admitting diagnoses).  Conventional care.  Routine medical care and nursing characteristic of an acute care unit. Team: nurses, geriatric physician, access to physiotherapist, occupational therapist, and social worker by means of referral, on consultation basis as needed. | Hospital admission and discharge. | Functional decline at discharge from baseline 2-week pre-hospital and hospital admission status, and length of hospital stay. |

^a^ Multiple studies refer to the same trial.

^b^ Usual care patients may have been transferred to another unit where they were residing at time of discharge.

^c^ Not all outcomes measured at all time points.

Appendix S3. Forest Plots

Iatrogenic complications

Falls


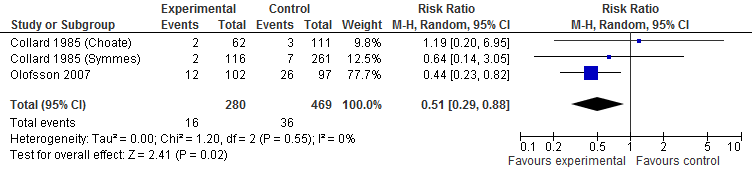


Pressure ulcers


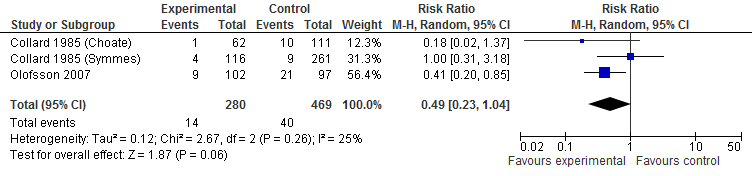


Delirium


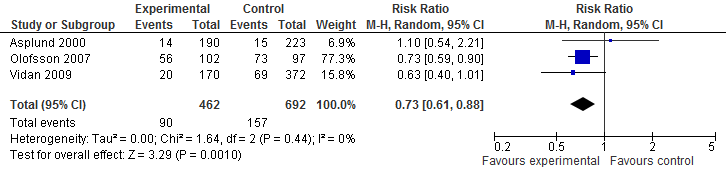


Functional decline at discharge from baseline

2-week pre-hospital admission status


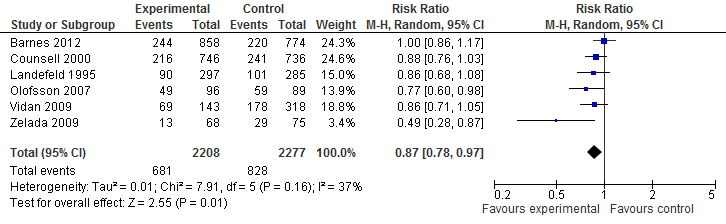


Hospital admission status


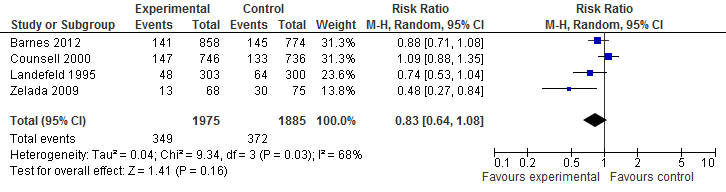


Hospital admission status – outlier removed


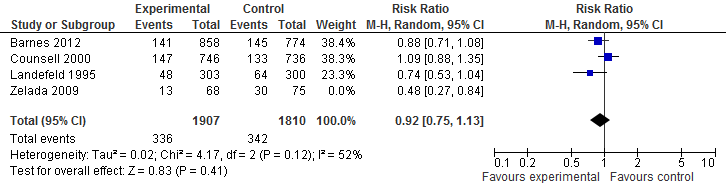


Length of hospital stay (days)


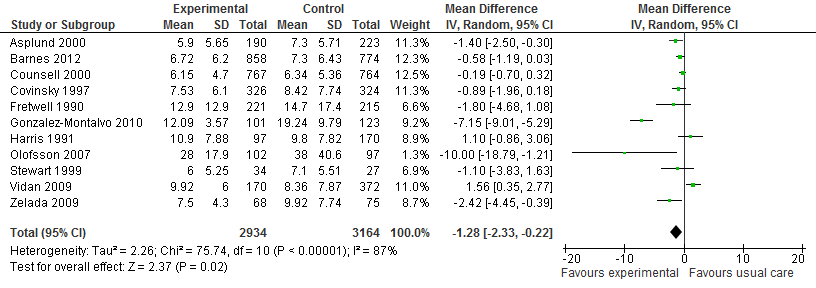


Length of hospital stay (days) -- Outliers removed


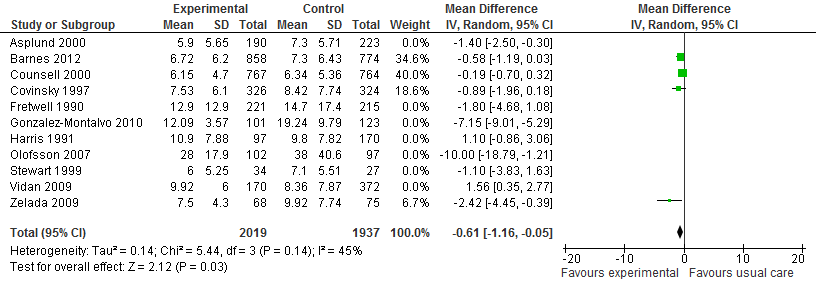


Discharge destination

Home


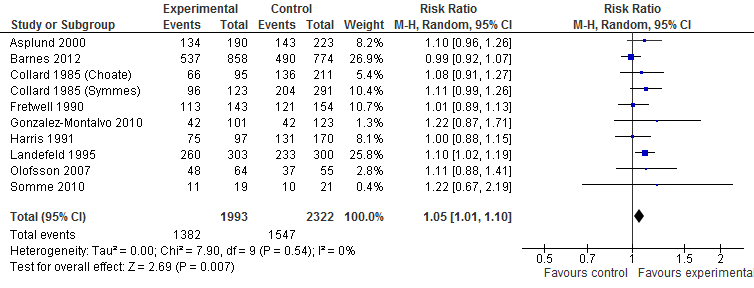


Nursing home


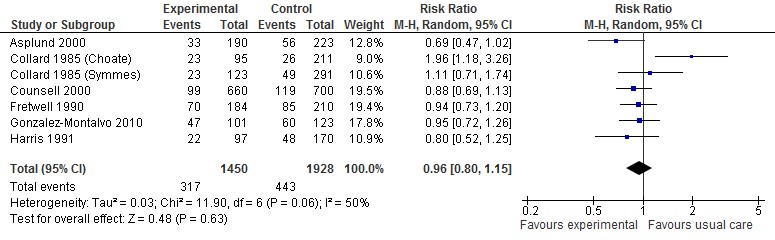


Nursing home -- Outliers removed


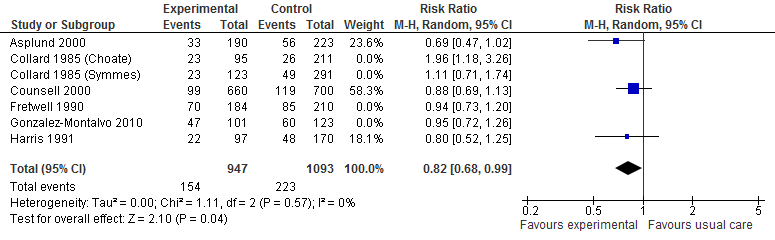


Mortality


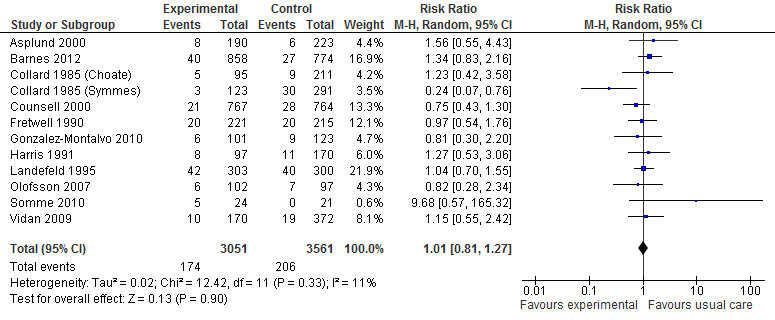


Costs


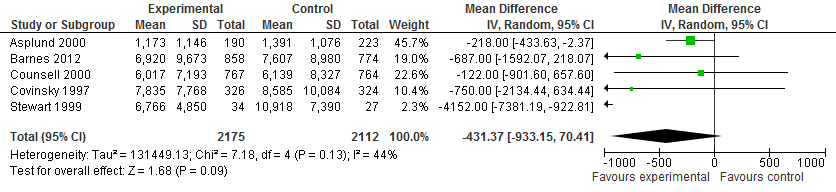


Costs -- Outlier removed


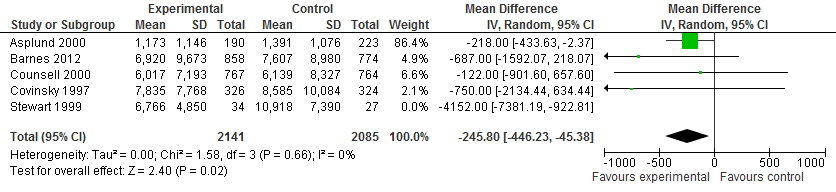


Hospital readmissions


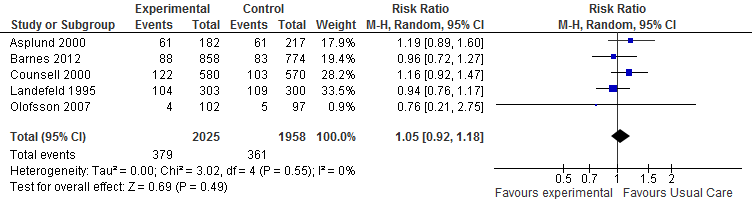

Supplement: Supplementary file 1 [file jgs0060-2237-SD1.docx]
